# Draft Genome Sequence of the Pyridinediol-Fermenting Bacterium *Synergistes jonesii* 78-1

**DOI:** 10.1128/genomeA.00833-14

**Published:** 2014-08-21

**Authors:** Hannah E. Holland-Moritz, David A. Coil, Jonathan H. Badger, George I. Dmitrov, Hoda Khouri, Naomi L. Ward, Frank T. Robb, Jonathan A. Eisen

**Affiliations:** a University of California Davis Genome Center, Davis, California, USA; b J. Craig Venter Institute, La Jolla, California, USA; c U.S. Army Center for Environmental Health Research, Fort Detrick, Maryland, USA; e University of Wyoming, Laramie, Wyoming, USA; f University of Maryland School of Medicine, Baltimore, Maryland, USA; g University of California Davis, Department of Evolution and Ecology, Department of Medical Microbiology and Immunology, Davis, California, USA

## Abstract

Here we present the draft genome of *Synergistes jonesii* 78-1, ATCC 49833, a member of the *Synergistes* phylum. This organism was isolated from the rumen of a Hawaiian goat and ferments pyridinediols. The assembly contains 2,747,397 bp in 61 contigs.

## GENOME ANNOUNCEMENT

*Synergistes jonesii* is a Gram-negative, anaerobic, non-spore-forming, nonmotile bacterium that was isolated from the rumen of a Hawaiian goat (1). *S. jonesii* has the capacity to ferment pyridinediols and was originally isolated for its suspected role in aiding Hawaiian ruminants grazing on *Leucaena leucocephala*, which is usually toxic due to 3,4-dihydroheptaprenol (3,4-DHP) produced in the digestion of the toxic amino acid mimosine (2). *S. jonesii* was selected in 2002 as part of a project at the Institute for Genomic Research to sequence the genomes of representatives of the seven phyla of bacteria that at the time had cultured representatives but no available genome sequence. The genome was sequenced with Sanger clone-based sequencing but the data were inadequate for high-quality assembly and the project was delayed pending additional sequencing with Illumina technology.

DNA was extracted using standard techniques. Sanger libraries (small and medium insert and fosmid) were constructed as previously described (3). Illumina paired-end libraries were prepared by sonicating DNA and using standard Illumina protocols and reagents (TruSeq).

A hybrid genome assembly was performed using MIRA (version 4.0) (4) with 28,530 Sanger reads (average length 1,020 bp) and 3,667,276 paired-end Illumina reads (length, 300 bp). The contigs resulting from this assembly were filtered to remove contigs shorter than 500 bp as well as those with <31% of the average coverage for the assembly. The assembly produced 61 contigs, and attempts to perform scaffolding with SSPACE (5) did not result in a higher-quality assembly. The final assembly contains 2,747,397 bp, with a GC content of 56% and an estimated coverage of 8.7× (Sanger) and 54× (Illumina).

Completeness of the genome was assessed using the Phylosift software (version 1.0.0_01). which searches for 40 highly conserved, single-copy marker genes (6). All 40 were found in this assembly. Automated annotation was performed using the RAST annotation server (7). The *S. jonesii* 78-1 genome contains 2,686 predicted coding sequences and 56 predicted RNAs.

### Nucleotide sequence accession numbers.

This whole-genome shotgun project has been deposited at DDBJ/EMBL/GenBank under the accession number JMKI00000000. The version described in this paper is version JMKI01000000. Complete Illumina reads have been deposited at the European Nucleotide Archive (ENA) with the accession number PRJEB6249. Sanger quality files are available at http://dx.doi.org/10.6084/m9.figshare.1009730 and Sanger reads are available at http://dx.doi.org/10.6084/m9.figshare.1009729.
